# With Our Editors

**Published:** 1907-01-15

**Authors:** E. C. Kirk


### WITH OUR EDITORS.

**LIMITATION OF DENTISTRY TO ITS MECHANICS.** The following excerpt is from a paper on "Errors in Dental Education," read before the Massachusetts Dental Society June 5th, 1906, and published in the *Dental Cosmos*, pages 731-739, by Eugene S. Talbot, M. S., D. D. S., M. D., L. L. D.-

"The establishment of a separate college conferring a separate degree narrowed dental teaching, and gradually but surely developed the mechanics of dentistry to their limit. The result is the profession—if it may be called such—view the lesions of the mouth only from the mechanic side. Students in most dental schools are taught that mechanics and operative dentistry are the principle departments, the other departments being minor studies. While the opposite is true, the result is that the student neglects these studies for branches seemingly likely to yield him most money after graduation.

The excuse of the dental student for not doing research work, writing papers, reading journals and books, is that "he is too busy or tired." Would not this be true of other profession judging by their hours of work?

"Imagine two students of equal ability: one studies medicine, the other dentistry. After completion of their course the graduate of medicine has broadened his knowledge of subjects generally, while the graduate of dentistry has narrowed his. The tendency of the teaching is toward the mechanic rather than the scientific. This then is the reason the dentist does not take journals, buy books and read then, do research work, and write original papers".

"Narrowed dental teaching;" Our good brother seems to have become wearied and depressed with much labor and faithful service, even so was it with the prophet of old;"And he said, I have been very jealous for the Lord God of host: because the children of Isreal have forsaken thy covenant, thrown down thy altars, and slain thy prophets with the sword and I, even I only, am left and they seek my life to take it away.And the Lord said unto him:Yet I have left me seven thousand in Isreal, all the knees which have not bowed unto Baal, and every mouth which hath not kissed him"

At the time of forming the first dental college there were but a few hundred dentists in this country, and but few of those could be called educated and refined gentlemen. To-day we have a coterie of highly educated members, hundred who would be a credit to any profession and thousands who are respected and a worthy part of the community in which they live.

Undoubtedly the average of scientific attainments in the profession is greater to-day than at any former period in the history of dentistry in this country: and we believe it is improving year by year. That the scientific, manipulative and social status of the average dentsit is not as high as the zealous leaders of our profession desire is true and it is well that it is so, otherwise there would be danger of stagnation of energies and retrogression.

Why should we lament the fact that the special college for teaching dentistry is narrowing? However, it is true that making a specialty of the dental aspect of medicine placed its votaries in a groove, thereby narrowing their field of vision. It is also true that confining oneself to a depart ment of dentistry (say prosthesis) is still more narrowing: but it is by concentration only that the greatest development of the profession is possible. The world has been evolved and made better only by the sacrifice of its leaders and teachers. Saint Paul recognized this fact when he said; "For I determined not to know anything among you, save Jesus Christ," &c.

Narrow—broad; Suppose we have a body of water one hundred one and one-half miles long, one and one-third mile wide and six inches deep. Would such a body of water be of any service to commerce? Only a breeding place for bullfrogs and anopheles. But it would be *broad*, placid and tranquil, and might be beautiful to look upon. Suppose we narrow this sheet of water to a canal twenty-six feet deep, seventy two feet wide at the bottom and two-hundred feet wide at the top, and we will have the Suez Canal upon which the commerce of the world can float.

"And gradually but surely developed the mechanics of dentistry to their limit",

No, not prosthesis, neither the mechanics, physics nor esthetics of prosthesis have anywhere near reached perfection, but if dentistry had not been narrowed to a special vocation it never would have been developed as it has been in the past sixty years.

We should not regret the narrowness of our profession; but rather that we have not developed it more, and that we have not greater mental and physical capacity for the good work—G. H. Wilson in November *Dentists Magazine.*

**CONCERNING ANESTHETICS IN GENERAL AND COCAIN IN PARTICULAR.**

This journal has always maintained a conservative attitude with respect to the administration of anesthetic agents in dental practice, and as a consequence has frequently expressed its disapprovalof the indiscriminate or careless use. It is not intended that the foregoing statement of our attitude toward the general question of the use of anesthetics in dentistry is to be interpreted as being in any degree antagonistic to the legitimate and intelligent use of these important agents. It is exclusively with respect to their misuse that our criticism is directed. It is mainly a question of where, when, and how to draw the line between the right and wrong use of these agents that directly concerns us.

The scientific study of physiology and pharmaco-dynamics has not as yet revealed the exact mechanism of anesthesia.

We do not know how the so-called anesthetic agents act in the production of their characteristic effect. The chemistry and physics of protoplasm, and its action with reference to its vital manifestations and relations, must needs be more intimately investigated and determined before we can clearly understand the action of drugs and be in position to more safely and certainly use them. But this much we do know about anesthetic and analgesic drugs, viz, that their action is in general terms, to paralyze the functional activity of the protoplasm of certain cells: the definite and marked sensory paralysis which they produce being their chief characteristic and that which gives to them their value as annihilators of pain.

The power of these agents to destroy pain by inducing a temporary sensory paralysis is, however, not by any means the limit of their paralyzing power. Increase of their action by the administration of doses larger in amount or spread over a prolonged period of time will ultimately bring about a corresponding motor paralysis, and, if the action be further intensified, paralysis of the involuntary muscular activities that control the cardiac and respiratory function, and lastly that total and final paralysis of all function which is the other name for death.

The factors involved are necessarily complex, and their interaction develops almost an infinity of material conditions to be met with in practice. The number of agents and combinations of agents now known and used for anesthetic purposes is comparatively large. Many of them are still decidedly within the experimental stage, for the reason that their mode of action is unknown and their dynamic factor has not been worked out. This multiplicity of agents and their almost limitless possible combinations creates in itself a large element of uncertainty in regard to the general problem. When we add to the foregoing the infinitely varied degree in which the human organism reacts towards the toxic effects of these preparations, how vital resistance is modified by heredity, by environment, by food habit, by disease conditions, by the temporary factors of traumatism, shock, etc., the conservative and thoughtful practitioner may well pause and take time to consider the seriousness of the problem before him when about to administer an agent which by the very nature of its action sends his patient a definite number of steps upon the road toward that bourn whence no traveler returns.

It is a moral question, an ethical problem of the first magnitude, which may be expressed in some such terms as, How far are we justified by the conditions presenting in dental practice in administering anesthetics for the relief of the pain factor? We admit at once that the use of anesthetics in dental practice has, *per se*, just as much warrant and justification as their use in any other department of the art of healing: but we wish to emphatically defend the proposition that the question of their use or otherwise is to be determined solely by the administrator upon the merits of the case, and not upon the whim, desire, or timidity of the patient. The fact that a patient "demands an anesthetic" can never be the justification for its use, and much less can the patient's choice of the agent be the warrant for its selection by the operator. "Because the patient wanted it;" "because the patient preferred this or that agent;" because a particular agent is or is not a "practice-builder" or a "money maker"—God save the mark!—are among the reasons given in recent dental literature by men who with the courage of their ignorance have thus sought to justify their use of certain anesthetic agents. Theirs is the type of mind that we have upon a former occasion referred to as the kind that would not have been benefited by listening to a course of lectures on ethics during their college career. It is a case not of immorality but amorality.

The choice of a particular agent is to be made upon the basis of its safety and of its adaptability to the case under consideration, both of which presuppose that the administrator is fortified with a knowledge of the nature of the agent which he selects, its mode of action, its limit of dose, its record of safety, and the antidotes and means for combating its dangerous effects; these on the one hand, and the physical condition and health status of his patient on the other. This applies to all cases where it has been decided that the induction of anesthesia is justifiable.

But there is the precedent question of whether the demand for painless dentistry by the use of anesthetic agents acting through the general circulation is a sufficient warrant for such general use of these drugs. The history of cocain and its allied preparations is an interesting and instructive one.

Much experimental use of cocain and incident loss of human life had to be gone through with before the reasonably safe physiological dose could with any approach to accuracy be determined. Moreover, it was found that an amount under one set of conditions could be safely injected would prove fatal under other conditions, depending apparently upon the relative areas of surface affected by the drug. The maximum physiological dose had to be determined and the minimum dose capable of producing an operable anesthesia as well. But with these factors fairly worked out it was nevertheless found that many cases of subsequent sloughing of the soft tissues ensued when cocain solutions had been injected previous to the extraction of diseased teeth.

The cause was sought in possible infection of the solution used, or of the hypodermic needle. Elimination of these possible sources of infection and surface sterlization of the territory of operation did not in all cases prevent subsequent necrosis of the soft tissues into which the drug had been injected. It has been found that sloughing is most prone to occur in cases where extraction is sought as the means of relief from pericemental inflammation in the acute stage, where the burden of the toxic action of the cocain is added to that generated in the tissues themselves by the pathogenic bacteria responsible for the inflammatory action which rendered the extraction of the tooth necessary. It is just such cases—those already poisoned by the end-products of the bacteria of inflammation, those in which the inflammatory action has already brought the soft tissues to the verge of necrosis—that an injection of cocain adds the further increment of toxicity necessary to destroy the cell vitality of the part and produces sloughing. Cocain in these cases is, we think, definitely contra-indicated.

We would go a step farther in criticism of the tendency to eliminate the pain element as a routine measure in all cases of dental treatment in operation. Frankly admitting that many dental operations are productive of a degree of pain which is beyond the limits of ordinary endurance, and that to inflict such a burden of pain is, or may be in many instances damaging to the nervous sytsem of a delicately organized patient, there is on the other hand much easily bearable suffering from dental operations for the relief of which an anesthetic is not only not needed, but where on moral ground we believe its use to be distinctively wrong. The moral point at issue is this: It is manifestly impossible to eliminate from the conditions of human existence the demand which because it panders to that weakness of nervous resistance which is the basis of inebriety, hysteria, low moral tone, and loss of vigor, which in its protean manifestations is so insidiously undermining the social fabric.

Somewhere we have seen recorded the case of a rich old dowager of the hysterical type, who employed a physician to travel with her in order that he might be at h nd to promptly administer chloroform to her when the train passed through tunnels, her nervous system not being capable of enduring the stress of the tunnel experience. We are in certain directions rapidly reaching a similar condition, and the dental profession is a contributory factor thereto in cocainizing humanity for every disagreeable sensation from which the hysterical or morally weak desire to escape. Pain within reasonable limits is a moral tonic, and as such is to be utilized just on the same principle as physical exercise, bodily work is utilized to develop a sound physical organization.—Dr. E. C. Kirk, December *Cosmos*.

